# Association Between Nutritional Status and Physical Activity Among Reproductive Age Women in Arba Minch Health and Demographic Surveillance Site, Southern Ethiopia

**DOI:** 10.3389/ijph.2025.1608161

**Published:** 2025-03-26

**Authors:** Darik Temesgen Assefa, Dessalegn Ajema Berbada, Melesse Mengesha Merkina, Desta Haftu Hayelom, Nigus Kabtu Belete, Simegn Wagaye Kefene, Befikadu Tariku Gutema

**Affiliations:** ^1^ School of Public Health, College of Medicine and Health Science, Arba Minch University, Arba Minch, Ethiopia; ^2^ Department of Public Health, Arba Minch College of Health Science, Arba Minch, Ethiopia

**Keywords:** nutritional status, physical activity level, reproductive age women, Arba Minch HDSS, Ethiopia

## Abstract

**Objectives:**

Reproductive age women in Ethiopia face significant double burden malnutrition. Although underweight prevalence has declined, overweight and obesity rates are raising due to rapid nutrition transitions and physical activity levels changes. This study aimed to assess the association between nutritional status and physical activity among these women.

**Methods:**

Community-based cross-sectional study was conducted in Arba Minch Health and Demographic Surveillance Site involving 422 randomly selected women from April to May 2022. Data were collected through interviews, including weight and height measurements. Multinomial logistic regression assessed associations, with significance at p < 0.05.

**Result:**

Approximately 20.1% women faced malnutrition with 7.8% underweight and 12.3% overweight/obesity. Notably, 84.6% engaging in ≥600 MET-minutes of physical activity weekly and inactive women were 2.8 times more likely to be overweight/obese. Significant associations were found between nutritional status and factors like educational status, contraceptive use, household food insecurity, and family size.

**Conclusion:**

Increased overweight/obesity risk among inactive women underscores the need for promoting active lifestyles and targeted interventions for better health.

## Introduction

The global population faces a double burden of malnutrition: which is the coexistence of under nutrition alongside over nutrition. Reproductive-aged women are also vulnerable to malnutrition [1]. Globally, in 2016, 15.4% of women of reproductive age were underweight and 39.2% were overweight and obese. Underweight is common among women in low-income countries, with the highest rates in southern and southeastern Asia, followed by sub-Saharan Africa. Conversely, overweight and obesity in the European Region, the Eastern Mediterranean Region, and American regions of America account for ≥50% [1, 2]. According to 2016 Ethiopia Demographic Health Survey data (EDHS), 22% of reproductive-age women were underweight and 8% women were overweight and obese [3]. The prevalence of underweight has declined over the last 16 years from 30% in 2000 to 22% in 2016. In contrast, overweight and obesity have increased from 3% in 2000 to 8% in 2016 [4].

Women of reproductive age are prone to being underweight owing to their high nutritional requirements. In developing countries, sex discrimination in food allocation may also contribute to women’s nutritional vulnerability. They are also vulnerable to overweight and obesity [5]. This double burden is now more prevalent in low-income and middle-income countries. This is due to rapid nutritional transition, characterized by major shifts in populations’ eating patterns and lifestyles. Engaging in less physical activity exposed women to overweight and obesity. On the other hand, excessive physical activity increases the number of calories your body uses for energy and result in low body weight [6–8].

Malnutrition independently has adverse effect on pregnancy outcomes. Women who are underweight before pregnancy are more likely to give birth to a low birth-weight baby, leading to preterm birth and poor fetal physical development [9, 10]. Being overweight or obese leads to adverse metabolic effects on blood pressure, cholesterol and triglyceride concentrations, and insulin resistance, which increase the risk of coronary heart disease, ischemic stroke, type 2 diabetes, polycystic ovarian syndrome, and breast cancer [2].

Since the prevalence of underweight is still high, and the prevalence of overweight and obesity is increasing, the nutritional status of women of reproductive age remains a major concern. However, there is limited evidence regarding association of nutritional status and physical activity level in Ethiopia. Hence, this study aimed to assess the association between nutritional status and physical activity level among women of reproductive age at Arba Minch Health and Demographic Surveillance Site in Southern Ethiopia.

## Methods

### Study Area and Period

This study was conducted from April to May 2022 at the Arba Minch Health and Demographic Surveillance System Sites (HDSS). The Arba Minch HDSS includes nine Kebeles from Arba Minch Zuria district (six kebeles) and Gacho Baba district (three kebeles). The kebeles included in this surveillance system were selected based on a number of factors that included size of population, attitude of the community, accessibility to services, and balancing urban and rural settings. The HDSS office is located in Arba Minch town, 505 km south of the capital city Addis Ababa. According to a 2021 report, there were 82,896 (41,772 females) populations; among them 21,665 were women of reproductive age whose age ranges 15–49 at the site. The area is well known for its cash crops, including bananas, mangoes, avocados, and vegetables.

### Study Design and Population

This was a community-based cross-sectional study. Reproductive-age women residing in the Arba Minch HDSS were included in the study and those who were pregnant and less than 6 months postpartum were excluded from the study.

### Sample Size Determination and Sampling Technique

The sample size was calculated using single population proportion formula through the assumptions of 95% Confidence Interval (CI), 5% margin of error, and 48.6% prevalence of underweight among reproductive age women from research conducted in Ziway Dugda district, Ethiopia [11], as this prevalence provides the largest sample size and aligns closely with the local population. Finally, considering 10% of nonresponse, the total sample size was 422. The study participants were selected using the Arba Minch HDSS database, which contains a list of all individuals in the nine kebeles, with their name, date of birth, sex, individual and household identification, pregnancy, and childbirth status. The sampling frame was developed by extracting the list of women of reproductive age for each kebele from the database, using sex and date of birth. The study participants were proportionally allocated to each kebele by considering the number of women of reproductive age and selected using simple random sampling technique which is computer-generated random numbers from each kebele, based on the sampling frame. The name and individual identification numbers were used to identify the women at their residency.

### Data Collection Instruments and Procedures

The data collection tool comprised both, standard tools and tools designed after an in-depth review and extraction from relevant literatures. In addition to the Arba Minch HDSS database for determining the status of women, a standard checklist developed by the Family Health International (FHI 360), a nonprofit organization focused on improving health and wellbeing, was used to check their pregnancy status during data collection [12]. Data were collected through structured and pretested interviewer-administered questionnaires by well-trained and experienced data collectors. The questionnaire included questions on socio demographic and economic characteristics, home garden availability, dietary habits, household food security, nutritional knowledge, and physical activity variables. A qualitative food frequency questionnaire which contains forty-eight food items commonly consumed in the study area was used to assess dietary diversity. Food security status was assessed using a standard complete form of Household Food Insecurity Access Scale (HFIAS), which was developed and validated by the Food and Nutrition Technical Assistant (FANTA) [13]. Physical activity levels were measured using a standard Global Physical Activity Questionnaire (GPAQ). Participants were asked if they had engaged in vigorous and moderate work and leisure-time activities continuously for at least 10 min. Transport related activities included only moderate-intensity activities worked out continuously for at least 10 min. They were asked the number of days engaging in each activity in a typical week, and the time spent in each activity in a typical day [14]. The weight and height were measured twice and the average value was calculated for reporting. Weight was measured using a digital floor scale (Model 869, Seca) to the nearest 0.1 kg without shoes, and height was measured using a locally made stadiometer to the nearest 0.1 cm with participants without shoes and headwear. The data collection tool was prepared and uploaded to Open Data Kit (ODK), a smartphone-based application for data collection.

### Operational Definitions

➢ Nutritional status: BMI was calculated using weight and height measurements (BMI = weight in meters divided by the square of height in meters). A BMI above 25 kg/m^2^ was categorized as overweight/obesity, and a BMI less than 18.5 kg/m^2^ as underweight.
➢ Dietary diversity: Using the Food and Agricultural Organization of the United Nations local food items were categorized into nine food groups [15]. The women’s dietary diversity score was calculated by summing the intake of the food groups over a period of 1 week. Participants were categorized based on the mean score of 6.2, those who scored above this mean value were considered to have a good dietary diversity score, while those who scored at or below the mean value considered to have a poor dietary diversity score.
➢ Physical activity level: The total time spent on physical activity during a typical week and the intensity of the physical activity metabolic equivalent (MET) minutes was calculated. Women who achieved 600 or more MET-minutes per week were considered as having sufficient physical activity, while those with fewer than 600 MET-minutes per week were considered to have insufficient physical activity [14].
➢ Vigorous-intensity activity: activities that make an individual breathe much harder than normal, such as forestry (cutting, chopping, carrying wood), intense farm work (plowing, manual tilling of soil, cutting crops), grinding with pestes or stones, and laboring.
➢ Moderate-intensity activity: activities that make an individual breathe somewhat harder than normal, such as brisk walking, domestic chores (cleaning, washing, cooking, milking cows, drawing water), moderate farm work (gardening, planting, weeding, harvesting crops, digging dry soil), weaving, tending animals, walking with load on head, wood work, and general building tasks (roofing and painting).
➢ Nutritional knowledge of the women on diet: The nutritional knowledge of women was categorized based on a mean value of 7.9. Women who scored above the mean score on the knowledge questions were classified as having “above mean score,” while those who scored at or below the mean as having “below mean score.”
➢ Household food insecurity: Nine occurrence and frequency of occurrence questions of FANTA 2007 were used to examine household food security scale [13]. Households were categorized into food secure and food insecure based on the household food insecurity access prevalence indicator. To quantify responses, the frequency of occurrence question is recoded as “0” if the answer for occurrence question is “No” while “Yes” responses were scored as follows: “1” for “rarely,” “2” for “sometimes,” and “3” for “often.” Food secure households scored 0 or 1 on the first question, and 0 to the rest 8 questions. Food insecure households scored 2 or 3 on the first question, and 1, 2 or 3 to the rest 8 questions.
➢ Formal education: Learning that occurs in a structured educational system, such as primary, secondary, or higher education institutions.
➢ Informal education: Learning that occurs outside a structured educational system.

### Data Quality Assurance

The English version questionnaire was translated in to “Amharic” and back into English to ensure consistency. Pretest was carried out on 5% of the study sample of those women of reproductive age, who were not selected as the study participants. To ensure data quality, data collectors were trained for 2 days about data collection method, ODK, and anthropometric measurements.

### Data Processing and Analysis

The collected data were downloaded from the ODK aggregate as a CSV file and exported to SPSS (version 25.0) statistical software for further analyses. Descriptive statistics such as frequencies, means, and standard deviation (SD) were used. Household wealth index was constructed using principal component analysis, considering durable household assets. This index divided into three categories (tertiles), and each household was assigned to one of these categories of household wealth index (low, medium and high). As the outcome variables had three categories: underweight, normal weight, and overweight/obesity, a multinomial logistic regression model was used to assess the factors associated with underweight and overweight/obesity by considering normal weight as the reference category. Bivariate analysis was used to determine the association between independent and dependent variables. Collinearity among the independent variables was assessed by measuring the variance inflation factors, and outliers were checked using a box plot. Variables with *p* value <0.25 were entered into a multivariate multinomial logistic regression model to assess the adjusted association between dependent and independent variables. The level of statistical significance was set at probability level of less than 0.05. The Adjusted Odds Ratio (AOR) along with and 95% confidence interval was used to assess the strength of association.

### Ethics Approval and Consent to Participate

Ethical clearance was obtained from the Institutional Research Ethical Review Board of Arba Minch University (IRB/1230/2022). Following approval, the official letter of cooperation was written to all nine kebeles, where the study was carried out to obtain permission to conduct the research. After randomly selecting the study participants, the Arba Minch HDSS provided the name, individual, and location identifications to the assigned data collectors. Data confidentiality was maintained in accordance with principles of the Declaration of Helsinki. Parental or guardian written informed consent was obtained from participants below the age of 18 years. Written informed consent was obtained from women above 18 years of age after explaining the objective of the study, risks and benefits, issues of confidentiality, and the right to participate. This study was approved by the Ethical review board of Arba Minch University.

## Results

### Socio-Demographic and Economic Characteristics of Women

A total of 422 reproductive age women participated in this study with response rate of 100%. The mean ± standard deviation (SD) age of the women was 27.1 ± 9.1 years. The majority of women were Gamo ethnic groups (80%), and more than half (56.4%) of them were married. Nearly two third (65.9%) of the women had formal education, and nearly half (46.9%) were housewives. Approximately half (56.2%) of the women were from family size greater than five (Table 1).

**TABLE 1:** Socio-demographic and economic characteristics of women of reproductive age in Arba Minch Health and Demographic Surveillance System Sites, Southern Ethiopia, 2022.

Variables		Nutritional status		
		Normal	Underweight	Overweight/obesity
Age	15–19	98 (23.2%)	12 (2.8%)	12 (2.8%)
	20–29	101 (23.9%)	5 (1.2%)	22 (5.2%)
	30–39	102 (24.2%)	7 (1.7%)	15 (3.6%)
	40–49	36 (8.5%)	9 (2.1%)	3 (0.7%)
Ethnicity	Gamo	268 (63.5%)	29 (6.9%)	41 (9.7%)
	Zeysie	40 (9.5%)	2 (0.5%)	4 (0.9%)
	Wolayita	22 (5.2%)	1 (0.2%)	4 (0.9%)
	Other	7 (1.6%)	1 (0.2%)	3 (0.7%)
Marital Status	Never married	129 (30.6%)	16 (3.8%)	19 (4.5%)
	Divorced	6 (1.4%)	2 (0.5%)	0
	Widowed	9 (2.1%)	0	3 (0.7%)
	Married	193 (45.7%)	15 (3.6%)	30 (7.1%)
Educational Status	Formal	215 (50.9%)	18 (4.3%)	45 (10.7%)
	No formal	122 (28.9%)	15 (3.6%)	7 (1.7%)
Occupational status	Housewife	155 (36.7%)	16 (3.8%)	27 (6.4%)
	Farmer	27 (6.4%)	1 (0.2%)	3 (0.7%)
	Daily laborer	25 (5.9%)	3 (0.7%)	1 (0.2%)
	Student	102 (24.2%)	11 (2.6%)	15 (3.6%)
	Merchant	17 (4%)	1 (0.2%)	2 (0.5%)
	Other	11 (2.6%)	1 (0.2%)	4 (0.9%)
Family size	<5	139 (32.9%)	14 (3.3%)	32 (7.6%)
	≥5	198 (46.9%)	19 (4.5%)	20 (4.7%)
House wealth index	Low	123 (29.1%)	16 (3.8%)	16 (3.8%)
	Medium	169 (40%)	12 (2.8%)	32 (7.6%)
	High	45 (10.7%)	5 (1.2%)	4 (0.9%)

### Dietary Habits and Nutritional Knowledge of the Women

The mean (SD) dietary diversity score of women was 6.2 (1.48). About three-fifths (60%) of women had poor dietary diversity score and 184 (43.6%) women were from households experienced food insecurity. Majority (96%) of the women typically ate a meal 3–4 times a day, and more than half (54.5%) of took an additional snack. Regarding knowledge of women, more than half (59.2%) of them had above mean score (Table 2).

**TABLE 2:** Dietary habits, home garden availability, nutritional knowledge and physical activity of women of reproductive age in Arba Minch Health and Demographic Surveillance System Sites, Southern Ethiopia, 2022.

Variables		Nutritional status		
		Normal	Underweight	Overweight/obesity
Dietary diversity	Poor	206 (48.8%)	22 (5.2%)	25 (5.9%)
	Good	131 (31.1%)	11 (2.6%)	27 (6.4%)
Household food insecurity	In secured	144 (34.1%)	22 (5.2%)	18 (4.3%)
	Secured	193 (45.7%)	11 (2.6%)	34 (8.1%)
Meal consumption per day	<2 times	4 (0.9%)	0	0
	3–4 times	325 (77%)	29 (6.9%)	51 (12.1%)
	≥5 times	8 (1.9%)	4 (0.9%)	1 (0.2%)
Habit of having snacks in a day	Yes	192 (45.5%)	14 (3.3%)	24 (5.7%)
	No	145 (34.4%)	19 (4.5%)	28 (6.6%)
Habit of skipping breakfast in a week	Never	277 (65.6%)	21 (5%)	44 (10.4%)
	1–4 days	46 (10.9%)	10 (2.4%)	7 (1.7%)
	≥5 days	14 (3.3%)	2 (0.5%)	1 (0.2%)
Habit of eating away from home in a week	Never	251 (59.5%)	25 (5.9%)	34 (8.1%)
	1–4 days	27 (6.4%)	3 (0.7%)	8 (1.9%)
	≥5 days	59 (14%)	5 (1.2%)	10 (2.4%)
Habit of eating fast foods in a week	Never	199 (47.2%)	24 (5.7%)	30 (7.1%)
	1–4 days	60 (14.2%)	3 (0.7%)	7 (1.6%)
	≥5 days	78 (18.5%)	6 (1.4%)	15 (3.6%)
Physical activity	Inactive	42 (10%)	8 (1.9%)	15 (3.6%)
	Active	295 (69.9%)	25 (5.9%)	37 (8.8%)
Nutritional knowledge	Below mean score	145 (34.4%)	17(4%)	10 (2.4%)
	Above mean score	192 (45.5%)	16 (3.8%)	42 (10%)
Home garden availability	Yes	64 (15.2%)	10 (2.4%)	8 (1.9%)
	No	273 (64.7%)	23 (5.5%)	44 (10.4%)

### Nutritional Status and Physical Activity of Reproductive Age Women

In general, the prevalence of malnutrition among women of reproductive age group in Arba Minch HDSS was 20.1% (95% CI: 16.43%–24.37%). The prevalence of underweight and overweight/obesity was 7.8% (95% CI: 5.42%–10.89%) and 12.3% (95% CI: 9.31%–15.84%), respectively. According to WHO recommendations on physical activity, 357 (84.6%) women spent ≥600 MET-minutes per week. Women aged 20–29 and 30–39 years engaged in sufficient physical activity with 32.2% and 31.1%, respectively. In contrast, women aged 40–49 years had the lowest engagement in sufficient physical activity with a participation rate of only 11.2%. In terms of occupational status, housewives led the adequate physical activity with 48.2% participation followed by students with 26.6%. Marital status was also a good indicator with married women participating most in sufficient physical activity at 59.1%, followed by never-married women with 35.9% participation.

### Physical Activity Level and Other Factors Associated With Nutritional Status

In multivariate multinomial logistic regression, women who had insufficient physical activity were 2.8 times more likely to be overweight than women who had insufficient physical activity (AOR = 2.78; 95% CI: 1.34, 5.78). There was no association found between physical activity level and underweight in this study.

In addition to these, as indicated in Table 3 the likelihood of underweight was significantly higher among women who did not use contraceptives (AOR = 3.26; 95% CI: 1.09, 9.71) and those from food insecure households (AOR = 3.04; 95% CI: 1.35, 6.82). The likelihood of overweight/obesity was significantly higher among women who attended formal education (AOR = 3.23; 95% CI: 1.37, 7.64). Women with a family size 5 and above (AOR = 0.43; 95% CI: 0.23, 0.81) were associated with decreased odds of overweight/obesity (Table 3).

**TABLE 3:** Physical activity and other factors associated with nutritional status of reproductive age women in Arba Minch Health and Demographic Surveillance System Sites, Southern Ethiopia, 2022.

		Underweight			Overweight/ Obesity		
Variables		Frequency (%)	COR	AOR (95% CI)	Frequency (%)	COR	AOR (95% CI)
Physical activity level	Insufficient Sufficient	8 (1.9) 25 (5.9)	2.25 1	2.74 (0.96, 5.87) 1	15 (3.6) 37 (8.8)	2.85 1	**2.78 (1.34, 5.78)** 1
Educational Status	Formal No formal	18 (4.3) 15 (3.6)	0.68 1	0.73 (0.33, 1.57) 1	45 (10.7) 7 (1.7)	3.65 1	**3.23 (1.37, 7.64)** 1
Family Size	<5 ≥5	14 (3.3) 19 (4.5)	1 0.95	1 0.94 (0.44, 2.03)	32 (7.6) 20 (4.7)	1 0.44	1 **0.43 (0.23, 0.81)**
Contraceptive use	No Yes	29 (6.9) 4 (0.9)	3.06 1	**3.26 (1.09, 9.71)** 1	29 (6.9) 23 (5.5)	0.53 1	0.59 (0.31, 1.15) 1
Household food insecurity	In secured Secured	22 (5.2) 11 (2.6)	2.68 1	**3.04 (1.35, 6.82)** 1	18 (4.3) 34 (8.1)	0.71 1	0.85 (0.44, 1.66) 1
Dietary diversity	Poor Good	22 (5.2) 11 (2.6)	1.27 1	0.96 (0.43, 2.14) 1	25 (5.9) 27 (6.4)	0.59 1	0.78 (0.41, 1.47) 1
Home garden availability	No Yes	23 (5.5) 10 (2.4)	0.54 1	0.56 (0.25, 1.26) 1	44 (10.4) 8 (1.9)	1.29 1	1.64 (0.69, 3.87) 1
Habit of having Snacks in a day	No Yes	19 (4.5) 14 (3.3)	1.80 1	1.48 (0.69, 3.15) 1	28 (6.6) 24 (5.7)	1.55 1	1.63 (0.88, 3.01) 1

The bolded ones indicates those with p-value < 0.05. COR, Crude odds ratio; AOR, Adjusted odds ratio.

## Discussion

This study revealed that the burden of malnutrition among women of reproductive age group in Arba Minch HDSS was 20.1%. The study provides evidence that among women of reproductive age in the Arba Minch HDSS, there is a double burden of malnutrition. Many other developing countries such as Malaysia, India, Palestine, Ghana, Mali, and Tanzania are also facing such problem [16–21]. Rapid shifts in dietary patterns and physical activity levels have led to an increase in overweight and obesity in low- and middle-income countries, which are already burdened by a high prevalence of underweight [22]. Additionally the study found that more than three-fourth (84.6%) of women had sufficient physical activity. The result is higher than the findings from previous studies conducted in Nepal [23] and Tanzania [20]. This discrepancy might occur due to difference in socio-economic and demographic characteristics difference.

The study found that insufficient physical activity was positively associated with being overweight or obese, but had no association with being underweight. This result aligns with findings from previous studies conducted in Nepal and Tanzania [20, 23]. A longitudinal study conducted in Australia further supported this finding by identifying critical periods in young women when high levels of physical activity protected against transitions to overweight and obesity [24]. Additionally, data from the National Nutrition and Physical Activity Survey (NNPAS) during the 2011–2012 Australian Health Survey revealed an inverse relationship between physical activity and BMI among postpartum women, with no association found in pre-pregnant women, while a study conducted in South Africa similarly indicated a negative correlation between physical activity index and BMI [25, 26]. This might be due to the fact insufficient physical activity causes an energy imbalance between calories consumed and expended [27]. The lack of association between physical activity level and underweight is also notable. This suggests that factors other than just physical activity, such as diet, metabolism, and underlying health conditions, likely play a larger role in determining underweight status. Physical activity alone does not necessarily lead to significant weight loss and an underweight.

Women who did not use contraceptive were 3.3 times more likely to be underweight than those who used contraceptives. This finding is consistent with that of studies conducted in the Oromia region of Ethiopia and Nigeria [28, 29]. Well-spaced births allow women to recuperate and replenish essential nutrients, leading to better nutritional outcomes [30]. In addition, women who do not use contraceptives are more likely to have short birth intervals between subsequent pregnancies, which can predispose them to being underweight.

Women from food insecure households were three times more likely to be underweight than those from food secure households. This result was similar to that of a study conducted in the Afar, Tigray, and Oromia regions of Ethiopia [11, 31, 32]. However, two studies conducted in Malaysia (Bachok District and Tuba Island) to assess the association between food insecurity and nutritional status using the Radimer/Cornell Scale (a tool for the assessment of food security status of households) showed no association between food insecurity and nutritional status [33, 34]. This opposition could be due to differences in the household food insecurity assessment tools used, which were the Radimer/Cornell Scale for studies in Malaysia and the HFIAS for this study.

Women with formal education were 3.2 times more likely to be overweight/obese than women without formal education. Similar results were reported in studies conducted in India and Palestine [16, 17]. In developing countries such as Ethiopia, educated individuals are more likely to consume energy-dense food and follow a sedentary lifestyle, which might be a reason for the increased prevalence of overweight/obesity among this group [35–37]. In addition, a systematic review of educational attainment and obesity showed that obesity and education are positively associated in lower-income countries [38].

Women with a family size of 5 or more were less likely to be overweight/obese than their counterparts. This might be because of women in high family size have many responsibilities, such as food preparation, agricultural activities, fetching water, and any other domestic chores. A large family size increases the work load of women, which can increase the energy requirement of the body. However, research conducted in Tanzania showed that family size was not associated with overweight/obesity [20].

These findings underscore the importance of promoting physical activity, especially among women, as a means of preventing overweight and obesity. Increasing opportunities for exercise and active lifestyles could have significant public health benefits in terms of weight management and reducing the risk of associated chronic diseases. The consistency of these results across different contexts highlights the generalizability of this relationship between physical activity and weight status.

### Conclusion

One in five women of the reproductive age group in the study area faced malnutrition, and both underweight and overweight/obesity were prevalent. Additionally, the prevalence of overweight/obesity was more than one in ten women. The prevalence of overweight/obesity was higher among women who had insufficient physical activity. This association that found between physical activity and overweight/obesity stress improving women’s physical activity helps to reduce overweight/obesity. Interventions are necessary to introduce community-based health promotion and education programs in order to promote awareness of the need for balanced nutrition and regular physical activity for women’s health. The programs should offer practical advice and support for healthier lifestyles. Additionally, Promoting comprehensive health services that incorporate nutritional education, food security assistance, and reproductive health services is essential since the factors are interlinked.

### Strengths and Limitations of the Study

Using multinomial logistic regression makes the study better to know the association between physical activity and nutritional status both underweight and overweight/obesity. The cross-sectional design of study limits the ability to make causal inferences about the relationship between nutritional status and physical activity level. To strengthen the causal inference, future studies should consider employing a longitudinal study design. Recall and reporting bias might also affect for dietary diversity and food frequency questions. To address this limitation, future studies should consider prospective method of dietary assessment. Additionally, this study lacks assessment regarding sex discrimination, and the evaluation of physical activity was based on only a questionnaire.

## Data Availability

Data will be available upon reasonable request from the corresponding author.

## Abbreviations

BMI, Body mass index; GPAQ, Global Physical Activity Questionnaire; HDSS, Health and Demographic Surveillance Site; HFIAS, House hold food insecurity access scale; ODK, Open Data Kit; SD, Standard deviation; WHO, World Health Organization.
